# Microscopy and supporting data for osteoblast integration within an electrospun fibrous network

**DOI:** 10.1016/j.dib.2015.10.009

**Published:** 2015-10-16

**Authors:** Urszula Stachewicz, Tuya Qiao, Simon C.F. Rawlinson, Filipe Veiga Almeida, Wei-Qi Li, Michael Cattell, Asa H. Barber

**Affiliations:** a Nanoforce Technology Ltd., Queen Mary University of London, Mile End Road, London E1 4NS, United Kingdom; b School of Engineering and Materials Science, Queen Mary University of London, Mile End Road, London E1 4NS, United Kingdom; c AGH University of Science and Technology, International Centre of Electron Microscopy for Materials Science and Faculty of Metals Engineering and Industrial Computer Science, Al. A. Mickiewicza 30, 30-059 Kraków, Poland; d Research Centre for Oral Growth and Development, Barts and The London Queen Mary׳s School of Medicine and Dentistry, 4 Newark Street, London E1 2AT, United Kingdom; e Centre for Adult Oral Health, Institute of Dentistry, Queen Mary׳s School of Medicine and Dentistry, Turner Street, Whitechapel, London E1 2AD, United Kingdom; f School of Engineering, University of Portsmouth, Portsmouth PO1 3DJ, United Kingdom

## Abstract

This data article contains data related to the research article entitled “3D imaging of cell interactions with electrospun PLGA nanofiber membranes for bone regeneration” by Stachewicz et al. [1]. In this paper we include additional data showing degradation analysis of poly(d,l-lactide-co-glycolide acid) (PLGA) electrospun fibers in medium and air using fiber diameter distribution histograms. We also describe the steps used in “slice and view” tomography techniques with focused ion beam (FIB) microscopy and scanning electron microscopy (SEM) and detail the image analysis to obtain 3D reconstruction of osteoblast cell integration with electrospun network of fibers. Further supporting data and detailed information on the quantification of cell growth within the electrospun nanofiber membranes is provided.

**Specifications table**

**Table:** 

Subject area	*Tissue engineering, Material Science, Biology,*
More specific subject area	*Electrospun nanofibers, FIB-SEM tomography, 3D imaging*
Type of data	*text file, graph, figure, movies, animations*
How data was acquired	*SEM, FIB-SEM ( Quanta 3D, FEI, E.U./U.S.A.)*
Data format	*Raw and analyzed data*
Experimental factors	*Electrospun samples after cell culture were FIB sectioned for 3D reconstruction. The reconstructed samples were analyzed for cell growth into the nanofiber network*
Experimental features	*- Degradation test of PLGA nanofibers in medium and air over 8 weeks time.- FIB-SEM tomography used for 3D visualization and quantification of cell integration with electrospun nanofibers*
Data source location	*UK, London,* Queen Mary University of London
Data accessibility	*Data is with this article*

**Value of the data**

• The data shows the successful application of FIB-SEM tomography of cells to 3D image cell-nanofiber interactions and quantification of cells growth within the network of nanofibers.
• The data provide examples of detailed analysis to verify cell growth at the filopodia level.
• The data provide proofs for cells integration within electrospun nanofiber networks despite network pore size being considerably smaller than typical cell diameter.
• Degradation studies of the electrospun nanofiber membranes over an 8 week time period shows reduction of membrane porosity and loss of fiber structure.

## 1 Data and materials and methods

### 1.1 Morphological analysis of PLGA fibers

Polymer solutions for electrospinning were prepared using poly(d,l-lactide-co-glycolide acid) (PLGA-lactide:glycolide (75:25), molecular weight: 66,000–107,000, Sigma Aldrich, U.K.) dissolved in a mixture of chloroform (analytical reagent grade, Fischer Scientific, U.K.) and N,N –dimethylformamide (DMF, 99.8%, Sigma Aldrich, U.K.) (85/15 mass ratio). Electrospinning of PLGA solution (15 wt%) was achieved using a single nozzle setup and a voltage of 14–15 kV and a distance 20 cm applied between the nozzle and a ground electrode. Degradation tests of PLGA in the form of electrospun nanofibers was carried out over 8 weeks in medium (Lonza Bio DMEM medium at 37 °C in a humid atmosphere under 5% CO_2_) and in air (dry conditions at room temperature (~22 °C) in darkness).

**Fig. 1:**
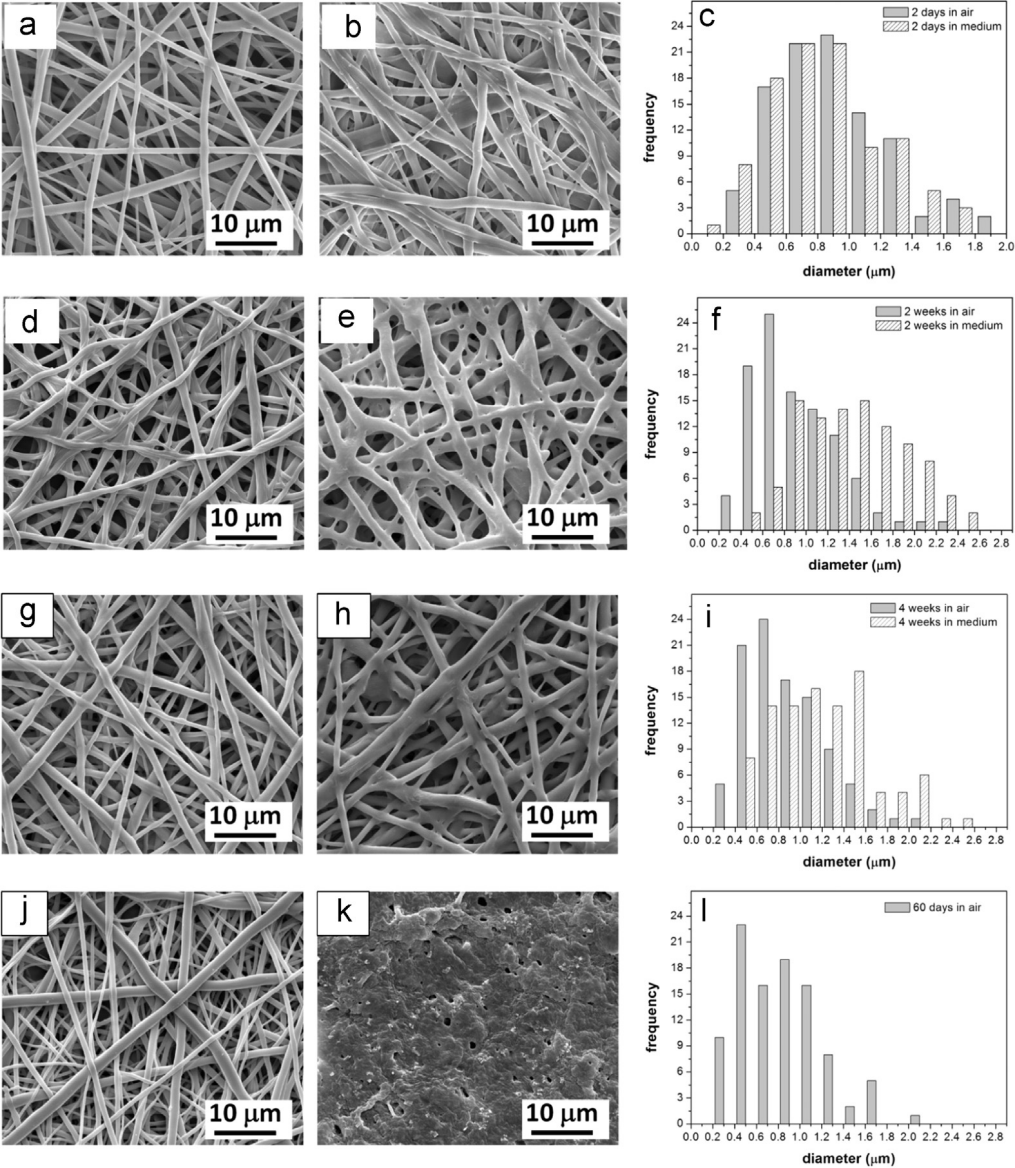
Scanning electron micrographs of electrospun PLGA nanofibers after degradation for (a) 2 days and (d) 2 weeks (g) 4 weeks (j) 60 days in air in darkness at room temperature (~22 °C) (b) in medium at 37 °C for (b) 2 days (e) 2 weeks (h) 4 weeks (k) 60 days; (c, f, i, l) fiber diameter size distribution histograms for degradation times.

The change in fiber diameter was recorded and the results are presented in the scanning electron microscopy (SEM) images and corresponding histograms in Fig. 1. Fiber diameter size distribution analysis was performed using image analysis (ImageJ, NIH, U.S.A.) with a total of 100 fiber diameter measurements taken from SEM images of each sample. For every image we set the scale bar so the fibers can be measured using the *Measure* function in the *Analyze* tab.

**Fig. 2:**
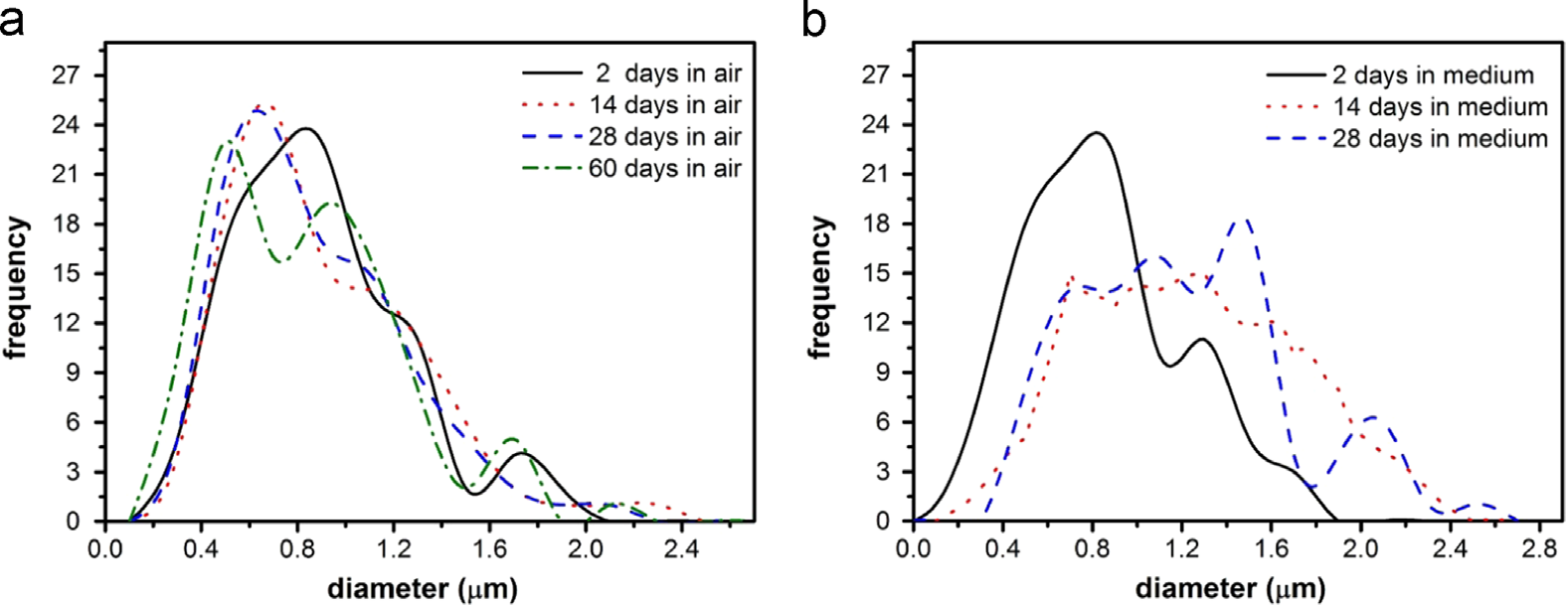
Diameter size distribution histograms examined over a range of timescales for the PLGA fibers samples kept (a) in air in darkness at room temperature (~22 °C) and (b) in medium at 37 °C.

A decrease in the PLGA fiber diameter kept in dry conditions was observed over 60 days of the test as shown in Fig. 2. Samples kept in medium increased their diameter and gave a wider fiber size distribution, resulting in pore size decreases over 28 days. After 60 days, the sample porosity was significantly reduced and fiber structures were difficult to identify for the size distribution measurements as shown in Fig. 1(k).

### 1.2 Scanning electron microscopy and focus ion beam

Cell seeding was performed prior to microscopy observation with 2 ml ( 450,000 cells per ml) of medium containing either rat osteoblast cell line (UMR 106, ATCC® CRL-1661™) or mouse cell line (MC3T3-E1, subclone 14, ATCC® CRL-2594™) that was added to each sample and cultured at 37 °C in a humid atmosphere under 5% CO_2_ for 4 days. Cells were cultured using Lonza Bio DMEM medium containing 4.5 g l-glutamine, 2% Fetal Calf Serum (FCS, Sigma–Aldrich, U.K.) for UMR 106 and 10% Fetal Bovine Serum (FBS, Sigma–Aldrich, U.K.) for MC3T3-E1, and penicillin/streptomycin (Invitrogen, U.K.) 100 units ml^−1^. Two microscope slides with electrospun PLGA nanofibers were placed per Petri dish.

**Fig. 3:**
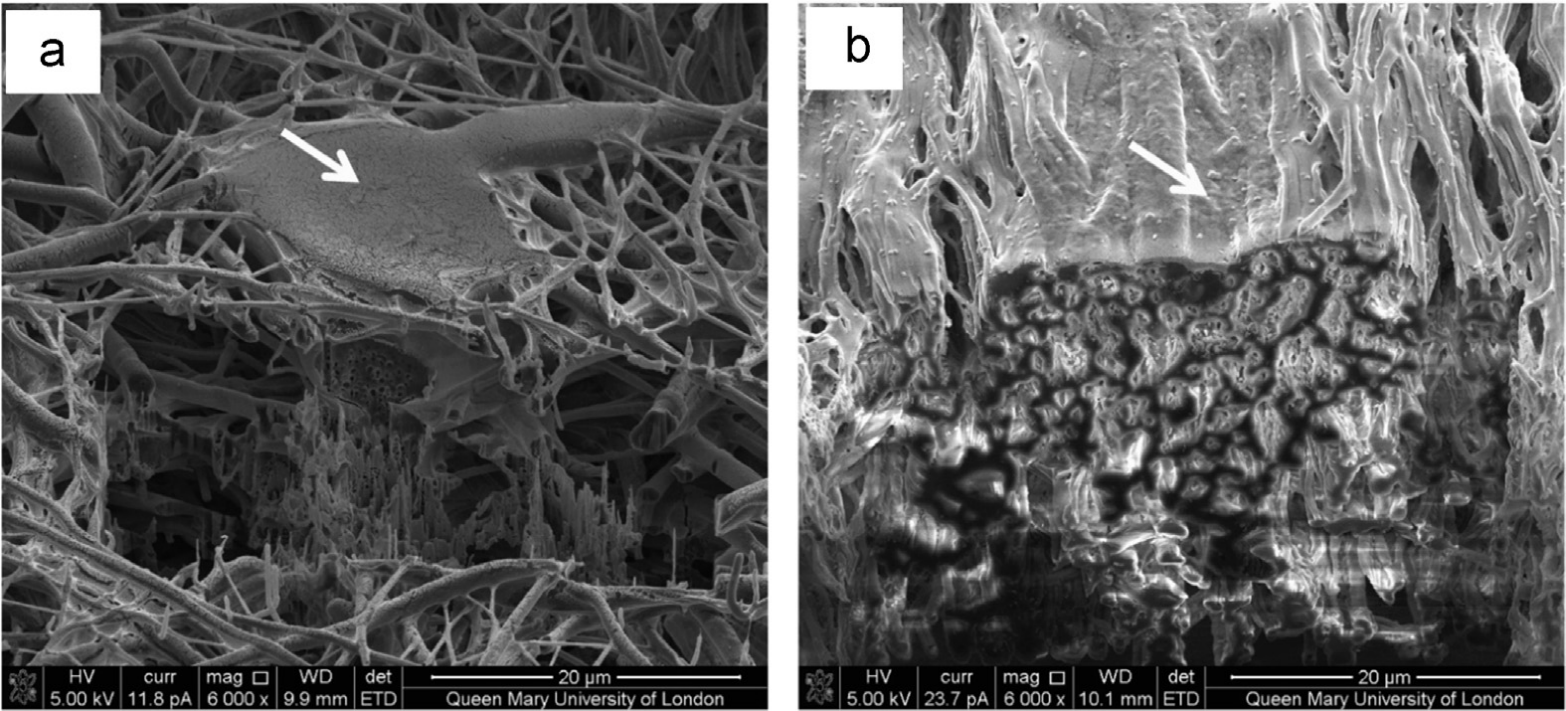
Scanning electron micrographs, taken at a 52° tilted stage view, of cross sections of (a) random and (b) aligned nanofiber samples with osteoblasts. Cells are indicated with arrows.

**Fig. 4:**
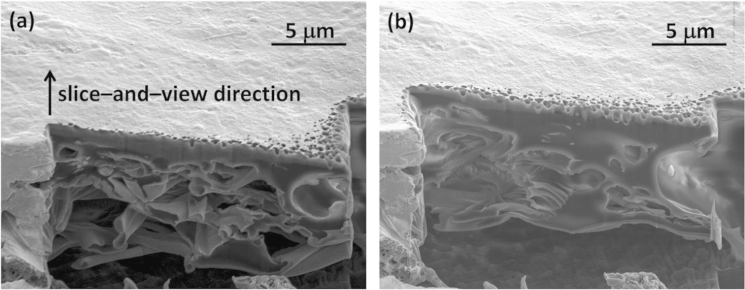
Scanning electron micrographs of electrospun PLGA networks with osteoblasts (a) before and (b) after sectioning the samples using FIB for 3D image reconstruction.

Imaging cell-nanofiber integration in random and aligned fibrous scaffolds, with SEM providing imaging of samples after subsequent focus ion beam (FIB) sectioning through samples, are presented in Fig. 3, Fig. 4. FIB sectioning was carried out following procedures defined in [2], [3] to minimize ion beam damage to samples. The video files of slice and view process are included in raw data files.

**Fig. 5:**
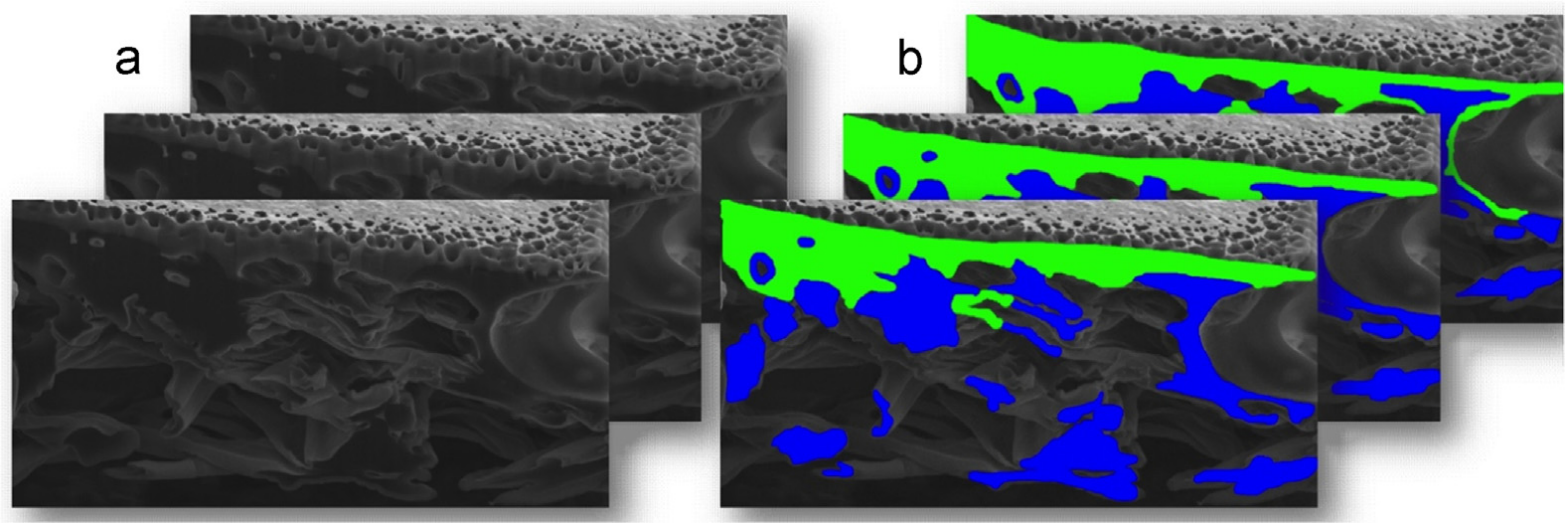
Examples of 2D image slices used for the 3D reconstruction of osteoblasts growing within an electrospun PLGA nanofiber network, including (a) a series of SEM images and (b) artificially colored images with osteoblasts shown in green and electrospun PLGA fibers in blue.

2D SEM images collected during FIB sectioning through a sample is shown in Fig. 5. Each 2D image was artificial colored using Image J (version 1.46r, NIH, USA) manually to differentiate osteoblasts from the electron nanofiber network. The collected 2D image data stack was used to create a 3D reconstruction as presented in [1].

**Fig. 6:**
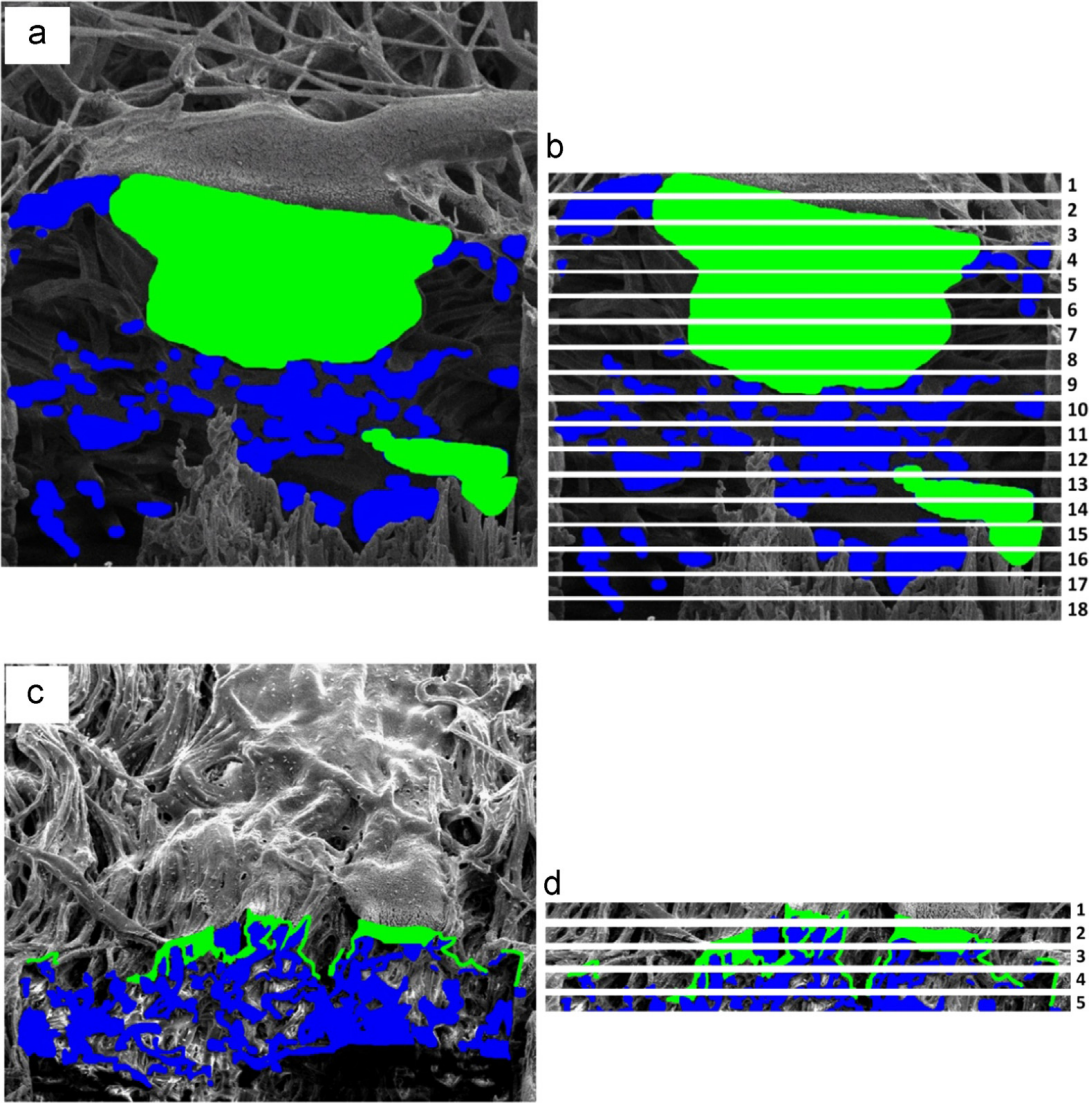
Artificially colored 2D scanning electron micrograph images showing a through-thickness cross-section in the *xz* plane for (a) a random electrospun nanofiber network and (b) subsequent slicing of the random fiber image into discrete layers up to 22 µm in depth for volume occupancy analysis (c) colored micrographs for an aligned fiber network and (d) subsequent layering of the image up to 5 µm in depth, which is smaller than for the random fiber case as cells are generally more surface limited, for volume occupancy analysis.

Analysis of volume occupied by osteoblasts within the electrospun nanofiber network was achieved by first digitally slicing membrane cross sections containing cells into 1 μm thick layers as indicated in Fig. 6. Image analysis was used to calculate the occupied volume of cells and nanofibers in each layer. Images were filtered using Image J by applying color thresholding and then exploiting the percentage area covered function on individual images to calculate volume composition. The volume occupancy of cells and fibers at a specific depth within the membrane, defined by the position of the layer considered, was therefore found and presented in Figure 10 of our studies [1].

### 1.3 Gene expression

**Fig. 7:**
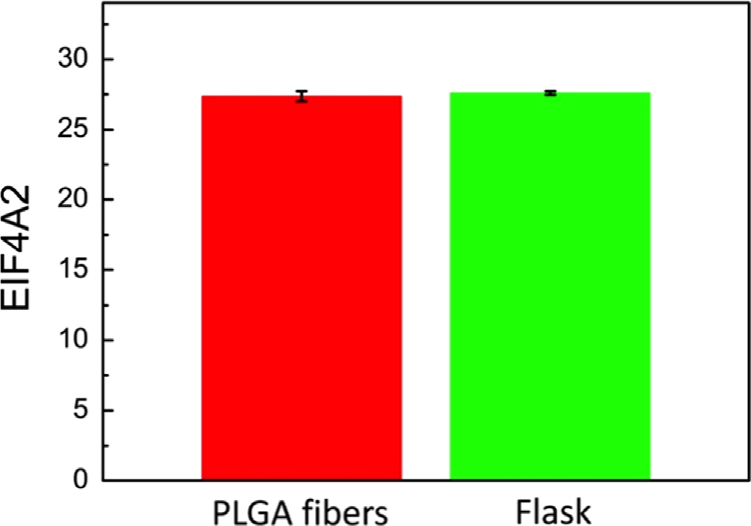
*Eif4A2* expression values stated as cycle threshold (Ct) for cells grown on PLGA fibers and plastic flask surfaces, demonstrating parity between the samples within standard error.

Western blot was used to determine protein production and qPCR applied for gene expression. Eukaryotic initiation factor 4A-II (EIF4A2) was used as a reference (house keeping) gene with Fig. 7 showing parity between gene expression results of PLGA fibers and flask samples.
